# Renal-Protective Urinoma Formation in a Newborn Boy With Posterior Urethral Valves

**DOI:** 10.7759/cureus.39880

**Published:** 2023-06-02

**Authors:** Nicole Alavi-Dunn, Kyle M Waisanen, Joseph A Marrara, Ash Zawerton, Ajit Monteiro, Kiana Saade, Ezekiel Young

**Affiliations:** 1 Urology, University at Buffalo, Buffalo, USA; 2 School of Medicine, Trinity School of Medicine, Kingstown, VCT; 3 Urology, Jacobs School of Medicine and Biomedical Sciences, University at Buffalo, Buffalo, USA; 4 Pediatrics, Wayne State University Detroit Medical Center, Detroit, USA

**Keywords:** urologic infection, urinoma, hydronephrosis, calyceal rupture, prenatal ultrasound, renal ultrasound, posterior urethral valves, pediatric urology

## Abstract

Posterior urethral valves (PUV) are an uncommon urologic congenital anomaly in males often discovered antenatally and more rarely after birth. PUV can lead to obstructive nephropathy and voiding dysfunction, putting patients at increased risk for irreversible renal damage and subsequent progression to end-stage renal disease. Much of the renal damage caused by PUV is proportional to the amount of time that the kidney has been experiencing retrograde pressure. Although much debate exists within the field, spontaneous decompression within the collecting system (e.g., “pop-off” valve) such as urinoma formation or spontaneous ascites has been found to relieve pressure on and thus protect the kidney, decreasing the risk of progression to advanced stages of chronic kidney disease. Despite the significant mass effect on the renal parenchyma, the pressure-relieving function of urinoma formation is a net protective factor allowing renal function to be preserved. We report a unique case of antenatal detection of PUV in a male with postnatal complicated urinoma formation secondary to forniceal rupture. Remarkably, despite significant external compression of the kidney and the development of urosepsis from infection of the urinoma with a multidrug-resistant organism that required percutaneous drainage, renal function was preserved throughout the disease course. After ablation of the PUV and drainage of the septic urinoma, the patient recovered rapidly after intervention and was ultimately discharged in stable condition.

## Introduction

Posterior urethral valves (PUV) are a congenital malformation in which membranous folds of tissue cause obstruction of the lower urinary tract at the level of the posterior urethra [1]. The origin is still unclear, but theories support abnormal Wolffian duct insertion into the urethra or persistent embryologic membranes [1]. PUV are exclusively seen in males. The incidence is 1 in every 5000 live births, and they are found on roughly 1/1250 antenatal ultrasound (US) screenings [2]. They are one of the most common pathologies leading to childhood renal failure and account for ~17% of children with end-stage renal disease [3]. The prognosis of PUV depends on the time at which the valves are suspected; if this occurs during the first trimester, they carry a very poor prognosis [4]. After the first trimester, there are better chances of survival, albeit commonly with compromised kidney function depending on the level of obstruction [3,4]. We report a unique case of PUV in which the resultant urinoma subsequently became infected after valve ablation and caused sepsis in the neonatal period.

## Case presentation

**Figure 1:**
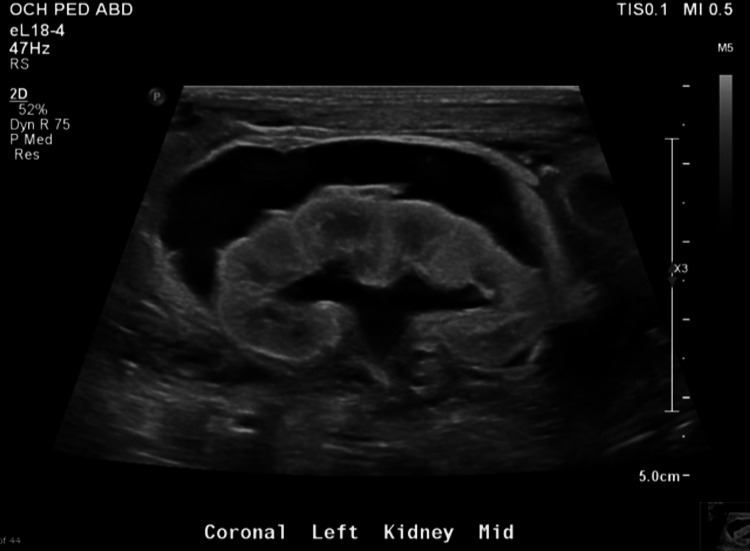
Ultrasound of left kidney on DOL1 demonstrating mild hydronephrosis, normal-appearing fetal renal parenchyma, and persistent left subcapsular fluid collection (initially seen on prenatal ultrasound). DOL: Day of life

**Figure 2:**
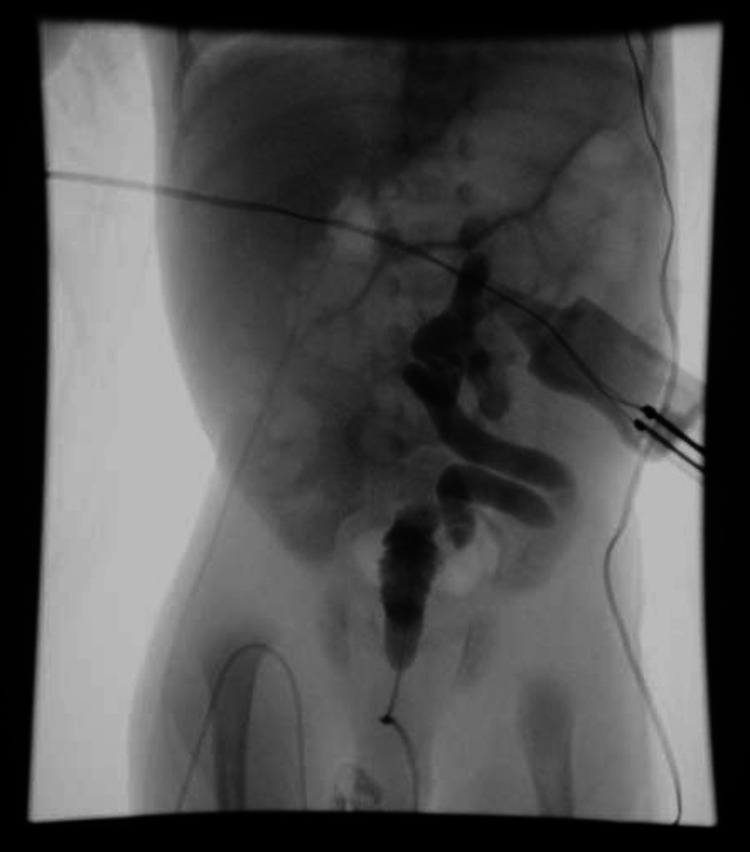
VCUG demonstrating small bladder volume with a dilated proximal urethra and grade 5 left vesicoureteral reflux. No extravasation noted. VCUG: Voiding cystourethrogram

A 33-year-old G3P1011 was referred to pediatric urology after third-trimester prenatal US detected fetal left hydronephrosis with a keyhole sign (thickened bladder with a dilated posterior urethra), suggestive of PUV. At the time, amniotic fluid levels were normal. On follow-up at 35 weeks gestation, repeat US demonstrated a new left subcapsular renal fluid collection and ascites, concerning for collecting system rupture. Concurrent findings of new oligohydramnios prompted admission by obstetrics for induction of labor. Failure to progress led to cesarean section delivery at 35w6d. The blood urea nitrogen/creatine (BUN/Cr) ratio at birth was 7/0.69. A 6-French feeding tube was placed per urethra to drain the bladder after birth due to concern for PUV. Renal bladder ultrasound (RBUS) performed post-delivery revealed bladder decompression, mild left hydronephrosis, normal fetal renal parenchyma, ascites, and persistent left subcapsular collection (Figure 1). The patient was started on prophylactic amoxicillin. Voiding cystourethrogram (VCUG) performed on day of life 7 (DOL7) showed small bladder volume with a dilated proximal prostatic urethra, compatible with PUV. It also revealed right grade 1 and left grade 5 ureteral reflux, with no obvious contrast extravasation (Figure 2). RBUS was repeated that day, with left subcapsular fluid collection now appearing complex and exerting a mass effect on the kidneys. The BUN/Cr ratio was 9/0.7. On DOL9, after receiving perioperative ampicillin, the patient underwent transurethral resection of clearly visible PUV via a cold knife. He was also found to have Hutch diverticulum on the left, with a retrograde pyelogram confirming the absence of left ureteral obstruction.

**Figure 3:**
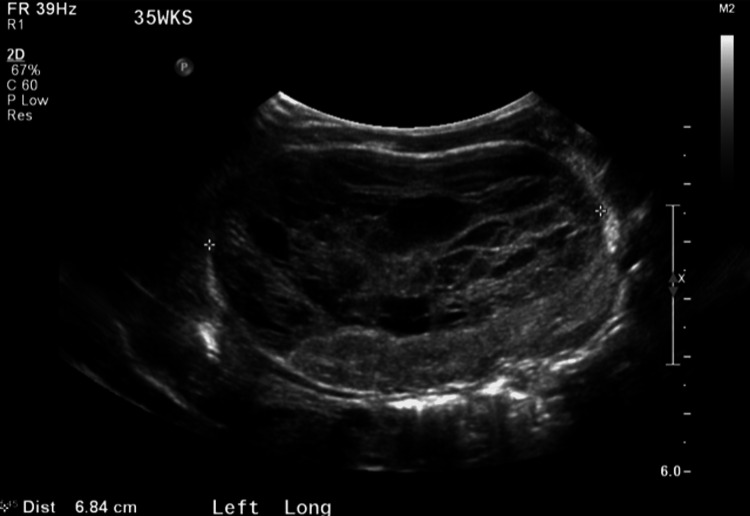
Ultrasound of the left kidney demonstrating septations within the perinephric fluid collection and compression of the kidney parenchyma.

**Figure 4:**
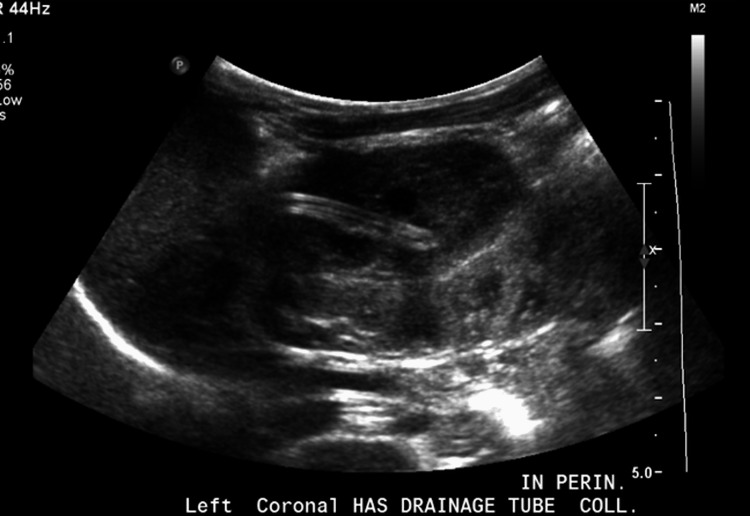
Ultrasound of the left kidney three days after a perinephric drain was placed. Note the decrease in the size of perinephric fluid collection with a drain in situ and decreased compression of the kidney parenchyma.

**Figure 5:**
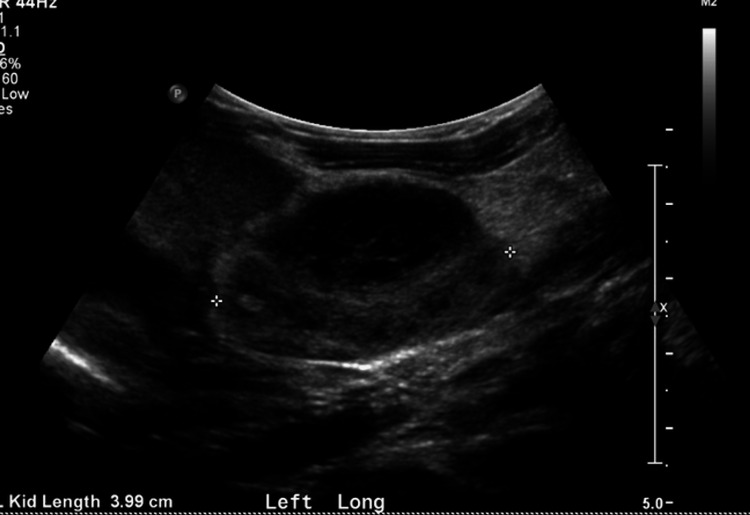
Ultrasound of the left kidney in the week after drain removal, demonstrating further decrease in the size of perinephric fluid collection and lack of hydronephrosis.

Repeat RBUS on post-operative day 4 (POD4) demonstrated persistent left subcapsular fluid collection with increased septations and absent hydronephrosis. The catheter was removed POD4 and spontaneous micturition followed. Labs on POD6 (DOL15) were suggestive of infection (leukocytosis 16.9, C-reactive protein (CRP) 78.65). RBUS revealed increased septations within the left perinephric fluid collection and reaffirmed lack of hydronephrosis in the left kidney (Figure 3). Broad-spectrum antibiotic treatment was initiated by the primary pediatric medicine team, with oxacillin and gentamicin. Infectious disease then recommended treatment with cefepime and gentamicin, discontinuing oxacillin. Urine cultures returned multidrug-resistant (MDR) Enterobacter cloacae; blood cultures were negative. For infectious diseases, cefepime was continued based on sensitivities, and gentamicin was discontinued. DOL18 labs demonstrated worsening signs of infection (leukocytosis 30.5, CRP 89.17). For source control, a US-guided 6.5 French drainage catheter was inserted into the left subcapsular fluid collection by Interventional Radiology. 20cc of yellow fluid was drained on initial placement; this also grew MDR Enterobacter cloacae. Three days later, RBUS confirmed a decrease in size of collection as well as decreased compression of kidneys (Figure 4). By the next day, leukocytosis improved to 19.9 with CRP 11.05. That day, the drain had nearly no further output and was removed. He continued to improve and was discharged the next week, having completed 14 days of cefepime. RBUS before discharge continued to show decreased fluid collection and no hydronephrosis as well as normal shape and echotexture of kidneys (Figure 5). The BUN/Cr ratio was 7/0.44. Circumcision was completed prior to discharge to decrease infection risk, and amoxicillin prophylaxis was restarted due to vesicoureteral reflux.

## Discussion

The gold standard for diagnosis during the antenatal period is routine US. The classical findings are bilateral hydronephrosis, distended bladder, and dilated proximal urethra with thickened bladder wall (together making the keyhole sign) [2,4]. PUV can be severe enough to include oligohydramnios causing pulmonary hypoplasia or have a milder delayed presentation as an older male with lower urinary tract symptoms such as incontinence and urinary tract infection [5]. Fetal urinary electrolytes can be used to estimate in-utero renal function; however, the predictive value of such studies remains limited [6]. In our patient, serial prenatal US was used as a diagnostic tool to evaluate urinary tract development in the fetus. Despite hydronephrosis and the suggestion of PUV, US demonstrated no concerns that would hasten prenatal intervention until the finding of oligohydramnios, which prompted the induction of labor.

Treatment modalities for PUV during the gestational period include the in utero open surgical technique of fetal vesicostomy, direct endoscopic valve resection, and vesicoamniotic shunting [7,8]. In 2013, the PLUTO (Percutaneous vesicoamniotic shunting in Lower Urinary Tract Obstruction) study results showed that post-natal survival was three times higher in the fetuses receiving vesicoamniotic shunting compared to conservative management; however, only two of seven shunted survivors had normal renal function at one year of age [9]. This showed that shunting increased survival but did not have a significant impact on long-term kidney function [3,9]. Because antenatal surgical treatments remain experimental without sufficient evidence of efficacy, active treatment for PUV is generally deferred until after birth [3]. Once the child is born, the first priority in the treatment of bladder obstruction is to relieve the upper urinary tract pressure by catheterization. Once stabilized, VCUG is the only direct investigation of the urethra in valve diagnosis. Most sources then recommend transurethral resection of the PUV since small enough instrumentation for such treatments is now widely available [3,5]. This is the course we followed with our patient.

PUV can lead to fatal kidney dysfunction due to the obstruction of urinary flow. A key event appears to be obstruction causing renal fibrosis antenatally, with multiple inciting factors including activation of the apoptotic and inflammatory cascade [10]. The decreased number of total nephrons present at birth leads to hyperfiltration injury, exacerbation of the underlying inflammatory process, renal fibrosis, and ultimately renal failure. In our patient, the constant increase in hydronephrosis led to the eventual rupture of the collecting system and resulting urinoma. We assume that the forniceal rupture acted as a “pop-off” valve, decreasing the upper tract pressure and thereby aiding in preservation of renal function. This has been similarly hypothesized in several case series [11-13]. Up to 20% of boys with PUV have extravasation of urine evidenced by urinomas or urinary ascites, either prenatally or neonatally [11]. These patients have a lower incidence of elevated nadir Cr and a lower prevalence of chronic kidney disease. In our patient, the extravasation of urine from forniceal rupture occurred in utero. This spontaneous decompression appears to have resulted in protection of renal function, as evidenced by BUN/Cr within normal limits and normal-appearing kidneys on ultrasound.

One study detailed urinoma drainage in several patients postnatally; in all cases, this was performed for respiratory distress. While our patient had no problems with respiration, he did have complications from his urinoma, developing infection causing sepsis. It is likely that the infection was introduced during instrumentation, either with catheter placement or cystoscopic procedure, despite perioperative antibiotics. When antibiotics and supportive care failed, the patient required drainage of his perinephric fluid collection, which had developed into loculated abscess. It is unclear why in his short life that had limited antibiotic exposure, the patient would develop MDR bacteria. In our literature search, we were unable to find any articles detailing infected urinomas and their subsequent management. One case series did uncover a patient who underwent nephrectomy at three months in a kidney with urinoma; this patient’s kidney was determined to be non-functioning, and the patient was suffering from recurrent urinary tract infections [12]. The other surviving patients in that case series had spontaneous resolution of urinoma. Although our patient did require intervention to recover from his infection, he improved quickly and was discharged in stable condition. With no admissions in the months after discharge, successful transurethral resection of his valves, and acceptable kidney function maintained, we will continue with close follow-ups to ensure the best outcome possible.

## Conclusions

In summary, early recognition and diagnosis of PUV are crucial to their management. After valve ablation, standard monitoring should include trending BUN/Cr and performing RBUS with VCUG as indicated. In the case of resultant urinomas, monitoring vitals and labs for signs of infection as well as obtaining serial RBUS may indicate impending complications. In addition, drainage of the urinoma should be considered when culture-specific antibiotics are not clearing the infection, as it could lead to better prognostic outcomes and preserved renal function.
